# Effect of LDL-Cholesterol Levels and Oral Atorvastatin on Outcomes After Pipeline Therapy for Intracranial Aneurysms

**DOI:** 10.1161/STROKEAHA.124.049833

**Published:** 2025-08-07

**Authors:** Xin Feng, Chi Huang, Xin Tong, Zhuohua Wen, Yajun Zhu, Mengshi Huang, Jiancheng Lin, Jiwan Huang, Hao Yuan, Anqi Xu, Gengwu Ma, Runze Ge, Can Li, Chao Peng, Shixing Su, Xin Zhang, Xifeng Li, Zongduo Guo, Aihua Liu, Chuanzhi Duan

**Affiliations:** Neurosurgery Center, Department of Cerebrovascular Surgery, Engineering Research Center of Diagnostic and Therapeutic Technology and Devices for Cerebrovascular Diseases, Ministry of Education, Zhujiang Hospital, Southern Medical University, Guangzhou, People’s Republic of China (X.F., C.H., Z.W., M.H., J.L., J.H., H.Y., A.X., G.M., R.G., C.L., S.S., X.Z., X.L., C.D.).; Department of Interventional Neuroradiology, Beijing Neurosurgical Institute, Beijing Tiantan Hospital, Capital Medical University, People’s Republic of China (X.T., A.L.).; Department of Neurosurgery, The First Affiliated Hospital of Chongqing Medical University, People’s Republic of China (Y.Z., Z.G.).; Department of Neurosurgery, Guangdong Provincial People’s Hospital, Guangzhou, People’s Republic of China (C.P.).

**Keywords:** atorvastatin, hydroxymethylglutaryl-CoA reductase inhibitors, intracranial aneurysms, ischemic stroke, lipoproteins

## Abstract

**BACKGROUND:**

We aimed to determine the effects of statin treatment on outcomes of pipeline embolization device therapy for intracranial aneurysms in relation to LDL (low-density lipoprotein) cholesterol levels.

**METHODS:**

Using data from the SESIA registry (Safety and Efficacy of Stent Deployment for Intracranial Aneurysms), we enrolled participants who underwent pipeline embolization device implantation at 4 centers in China (2018–2022). Statin users (atorvastatin 20 mg daily, for ≥3 days and ≥6 months preprocedurally and postprocedurally, respectively) were matched with nonstatin users (1:1) using propensity scores and further adjusted by inverse probability of treatment weighting, balancing for baseline characteristics, procedural details, and lipid levels (based on East Asian profiles). Study outcomes include perioperative complications, aneurysmal occlusion, in-stent stenosis, and clinical prognosis at the latest follow-up. Multivariable analyses using logistic and Cox regression models adjusted for these factors in both prematched and postmatched cohorts, evaluating the role of lipid modification in subgroups.

**RESULTS:**

Of the 1558 patients screened, 1193 (53.75±11.07 years, 69.4% females; statin: n=603, nonstatin: n=590) were enrolled with 3- to 48-month follow-up. In the matched cohort (352 pairs), statin treatment reduced the incidences of perioperative cerebrovascular (2.0% versus 8.5%, *P*=0.001) and follow-up ischemic (1.7% versus 5.1%, *P*=0.020) complications. Multivariable analyses in participants with baseline LDL-cholesterol ≥2.59 mmol/L showed that statin treatment was associated with fewer perioperative cerebrovascular complications (odds ratio, 0.371 [95% CI, 0.195–0.705]; *P*=0.002), in-stent stenosis (hazard ratio, 0.433 [95% CI, 0.269–0.698]; *P*=0.001), and follow-up ischemic events (hazard ratio, 0.315 [95% CI, 0.135–0.733]; *P*=0.007; *P* values for interaction were 0.671, 0.009, and 0.507, respectively, versus <2.59 mmol/L). Postmatched analyses confirmed consistency for perioperative cerebrovascular complications (odds ratio, 0.416 [95% CI, 0.211–0.821]), in-stent stenosis (hazard ratio, 0.397 [95% CI, 0.232–0.667]), and follow-up ischemic events (hazard ratio, 0.364 [95% CI, 0.148–0.895]).

**CONCLUSIONS:**

Atorvastatin treatment improved postpipeline embolization device-deployment outcomes by reducing ischemic events, particularly in patients with elevated LDL-cholesterol. The long-term benefits of adjunctive statin use in this population warrant further investigation.

**REGISTRATION:**

URL: https://www.clinicaltrials.gov; Unique identifier: NCT03387995.

Intracranial aneurysm (IA) rupture is a predominant cause of subarachnoid hemorrhage, a subtype of stroke with a high fatality rate and residual neurological deficits.^1^ Advanced neurointerventional devices, including flow diverters (FDs), such as the pipeline embolization device (PED; Medtronic, Minneapolis), are the mainstay of the endovascular therapeutic strategy for IAs, with occlusion rates of 75% to 85%.^2^ Despite their wide application, the overall complication rate (12.8%) and in-stent stenosis (ISS) rates (10% to 20%) that are associated with PED remain a concern.^3,4^

Elevated LDL (low-density lipoprotein) cholesterol levels increase the risk of vascular events,^5^ for which 3-hydroxy-3-methylglutaryl-coenzyme A reductase inhibitor (statin) therapy constitutes a cornerstone of prophylactic management. The therapeutic advantages of statins potentially exceed their traditional effect on lowering cholesterol levels and include additional pleiotropic effects, such as anti-inflammatory, vasodilatory, anticoagulant, platelet inhibitory, and antioxidant activities, many of which occur within 24 hours of initiating statin therapy and may mitigate the multiorgan effect of perioperative surgical stress.^6^ Thus, statin administration is recommended to improve surgical outcomes in various vascular conditions.^7–10^ However, it remains unclear whether these pleiotropic effects influence the outcomes of flow-diversion treatment for IAs.

Although several pharmacological studies have demonstrated the beneficial effects of statins on IAs,^11,12^ the relationship between statin treatment and post-PED outcome remains controversial.^13,14^ In a sample exclusively comprising statin users, the disparities in statin type, dosage, oral duration, and relevant LDL-cholesterol levels warrant further exploration of the effectiveness of statin treatment.

Therefore, we investigated whether standard statin treatment was associated with improved outcomes and whether these effects were modulated by the LDL-cholesterol levels.

## Methods

### Participants

This retrospective analysis used data from a prospective multicenter registry of consecutive patients with IAs in the Chinese population: SESIA (Safety and Efficacy of Stent Deployment for Intracranial Aneurysms). From the SESIA data set, we identified patients who received PED therapy between January 2018 and December 2022. The major exclusion criteria are as follows: (1) ruptured aneurysms; (2) previous embolization or neurosurgical treatment for target aneurysms; (3) tandem treatment (2 or multiple IAs covered by a single PED); (4) lacking information on fasting lipid profiles at hospitalization; (5) presence of other cerebrovascular abnormalities (ie, significant cerebrovascular atherosclerotic stenosis, arteriovenous malformations, arteriovenous fistulas, and moyamoya disease); (6) use of other types of statin prescription (ie, rosuvastatin, simvastatin, pravastatin); (7) lipid-lowering medications other than statins (ie, fibrates, ezetimibe, pcsk9 inhibitor); and (8) adverse events owing to improper operation during the procedure (ie, arterial perforation, failed stent deployment).

### Data Availability Statement

Anonymized data supporting the findings of this study are available upon reasonable request to the SESIA Steering Committee (13681134001@163.com).

### Ethics Statement

The local institutional review board of Zhujiang Hospital approved (number: 2017-SJWK-002) the utilization of clinical information from the SESIA registry. Written informed consent was obtained from all patients included in the registry. Anonymized data supporting the findings of this study are available upon reasonable request to the SESIA Steering Committee. This study adhered to the Strengthening the Reporting of Observational Studies in Epidemiology guidelines for cohort studies.

### Data Collection

Demographic characteristics and aneurysmal imaging data were prospectively collected (Supplemental Material). Fasting lipid profiles were obtained during the first 24 hours after admission.

### Procedural Details

The procedural management for PED implantation is described in the Supplemental Material.

### Definition of Statin Treatment

In our cohort, the statin that was prescribed was atorvastatin (Lipitor, Pfizer Pharmaceuticals, NY). As pleiotropic effects occur within 24 hours of statin intake,^6^ we defined statin users as patients who either received statins continuously before admission and continued statin therapy for at least 6 months postoperatively, or initiated statins at least 3 days before FD placement and maintained use for at least 6 months postoperatively.

### Levels of Baseline LDL-Cholesterol

Baseline LDL-cholesterol stratification was scaled as <2.59, 2.59–3.36, 3.36–4.14, and ≥4.14 mmol/L, in accordance with the phenotypic characteristics of serum lipids in the East Asian population.^15^

### Study Outcomes

The primary outcomes included perioperative cerebrovascular complications and new-onset cerebrovascular complications during follow-up, incidence of ISS, and composite cerebrovascular events. Cerebrovascular complications included ischemia, hemorrhage, and death during the perioperative period. Perioperative hemorrhages included intraoperative rupture, postoperative subarachnoid hemorrhage, and intraparenchymal hemorrhage.^4^ Perioperative ischemia was defined as follows: (1) transient ischemic attack; (2) mild ischemia (National Institutes of Health Stroke Scale score change ≤4, lasting <7 days, with corroborative imaging); and (3) severe ischemia (National Institutes of Health Stroke Scale score change >4, lasting >7 days).^16^ Composite events included new-onset cerebrovascular complications, unfavorable functional prognosis (modified Rankin Scale score >2), and all-cause mortality during follow-up. ISS refers to >25% narrowing of the FD diameter without preexisting significant artery stenosis.^4^

The secondary outcome was the aneurysmal occlusion status at the last follow-up that was evaluated according to the O’Kelly Marotta classification^17^: complete occlusion (D, entirely nonfilling) and incomplete occlusion (A, total filling; B, subtotal filling; and C, entry remnant). These assessments were supervised by 3 neurointerventionalists with >20 years of experience, and, in cases with discrepant assessment results, a discussion-based unanimous decision was followed.

### Follow-Up Protocol

The follow-up protocol has been previously reported.^4^ Briefly, mandatory digital subtraction angiography was performed at the 3- or 6-month follow-up postprocedure. For incomplete occlusions, subsequent angiographic checks were scheduled every 6 to 12 months, utilizing digital subtraction angiography (preferred), computed tomography angiography, or magnetic resonance angiography. Clinical follow-up was performed for all patients, regardless of imaging availability, and was conducted by in-person consultations, video calls, or telephone calls. The first clinical follow-up was conducted within 30 days of discharge and repeated every 6 months thereafter, which may not have been conducted simultaneously with angiographic follow-up. All follow-up intervals were determined from the most recent archived follow-up date.

### Statistical Analysis

Baseline characteristics of the statin and nonstatin groups were compared using the independent *t* test or Wilcoxon rank-sum test for continuous variables, and the χ^2^ or Fisher exact test for categorical variables, as appropriate. Covariate balance before and after propensity score (PS) matching was evaluated using absolute standardized differences, with absolute standardized differences <0.1 indicating satisfactory balance. The PS was estimated for each patient using a multivariable logistic regression to predict the probability of receiving peri-procedural statin therapy. Covariates were systematically selected based on clinical relevance (age, sex, body mass index, hypertension, diabetes, ischemic history, coronary artery disease, smoking status, alcohol consumption, and baseline LDL-cholesterol level), lesion-specific parameters (aneurysm location [anterior/posterior], size, and morphology [saccular, dissecting, or fusiform]), and procedural factors (adjunctive coils, balloon angioplasty, and overlapping-stents technique).^3,4,13,16,18–22^

The primary analysis was constructed using 1:1 nearest-neighbor PS matching without replacement and a caliper width of 0.2 of the SD of the logit of the PS.^10,15^ Multivariable logistic regression for binary perioperative outcomes (ie, cerebrovascular complications, ischemic, hemorrhage). Cox proportional hazard regression for time-to-event outcomes (ie, ISS, clinical events, and composite end points) during angiographic follow-up (mean: 17.00±8.41 [range, 3–42] months) and clinical follow-up (mean: 25.83±6.96 [range, 12–48] months). Each multivariable model was adjusted for covariates based on clinical relevance and literature review. A treatment-by-subgroup interaction term (statin treatment×LDL-cholesterol threshold <2.59 versus ≥2.59 mmol/L) was included to assess effect modification, in conformance with recent dyslipidemia guidelines.^5,23^

As a sensitivity analysis, inverse probability of treatment weighting was applied by weighting the inverse probability of a patient based on the PS to create a pseudopopulation. This model preserved the size of the cohort without removing any participants, offered advantages over the PS matching analysis method, and reduced the occurrence of false positives as compared with inverse probability of treatment weighting. Moreover, weighted logistic and Cox models were constructed to confirm the robustness of the results in multivariable analyses of the PS-matched cohort. Weighted Kaplan-Meier curves and log-rank tests were used to compare time-to-event outcomes between treatment groups. Statistical significance was defined as a 2-sided *P*≤0.05. All analyses were performed using R version 4.2.0 (R Foundation for Statistical Computing, Vienna, Austria).

## Results

### Participant Characteristics

**Figure 1.**
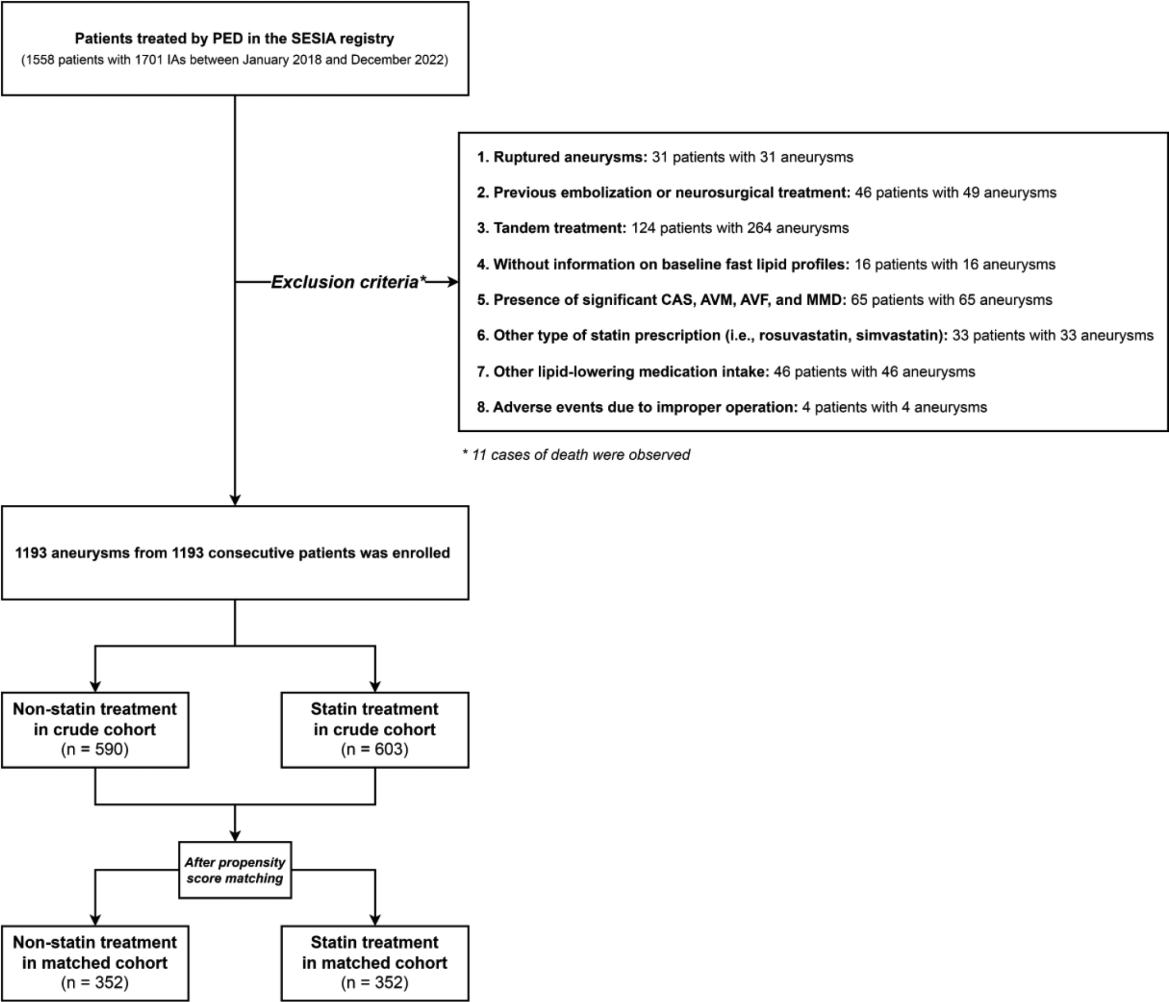
**Flowchart showing participant inclusion and exclusion in the SESIA clinical trial (Safety and Efficacy of Stent Deployment for Intracranial Aneurysms).** AVF indicates arteriovenous fistula; AVM, arteriovenous malformation; CAS, cerebral arterial stenosis; IA, intracranial aneurysm; MMD, moyamoya disease; and PED, pipeline embolization device.

A total of 1558 participants were initially screened. After the application of the exclusion criteria, 1193 patients (age, mean±SD: 53.75±11.07 years; n=603 and n=590 in the statin and nonstatin groups, respectively) met the eligibility criteria. A detailed patient selection flowchart is shown in Figure 1. Compared with the nonstatin treatment group, the statin treatment group was more likely to include participants who were older and had a history of diabetes and ischemia, higher baseline LDL-cholesterol levels, and nonsaccular morphology and was less likely to include patients with higher body mass index, alcohol consumption, and larger aneurysm size (all absolute standardized differences >0.10). After PS matching, the baseline characteristics of the statin and nonstatin treatment groups were well-balanced (Table 1). Figure S1 shows the distribution of PS.

**Table 1.**
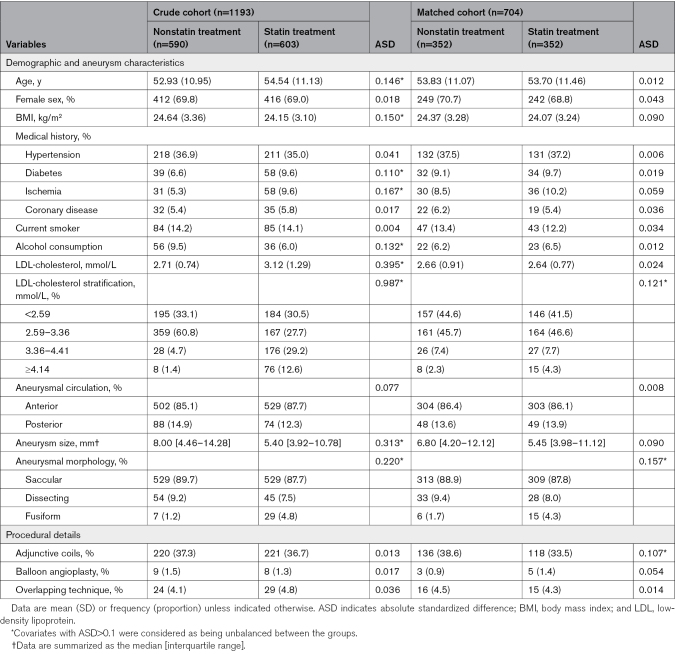
Demographic Characteristics, Aneurysmal Features, and Procedural Details

### Perioperative and Follow-Up Outcomes

As shown in Table 2, statin therapy significantly mitigated the incidence of perioperative complications (4.0% versus 8.1%, *P*=0.003) with a pronounced reduction in postoperative ischemia (2.2% versus 5.8%, *P*=0.002). These beneficial effects were sustained in the propensity-matched cohort, where statin use was associated with lower rates of perioperative complications (2.0% versus 8.5%, *P*<0.001) and postoperative ischemia (1.1% versus 6.0%, *P*=0.001).

**Table 2.**
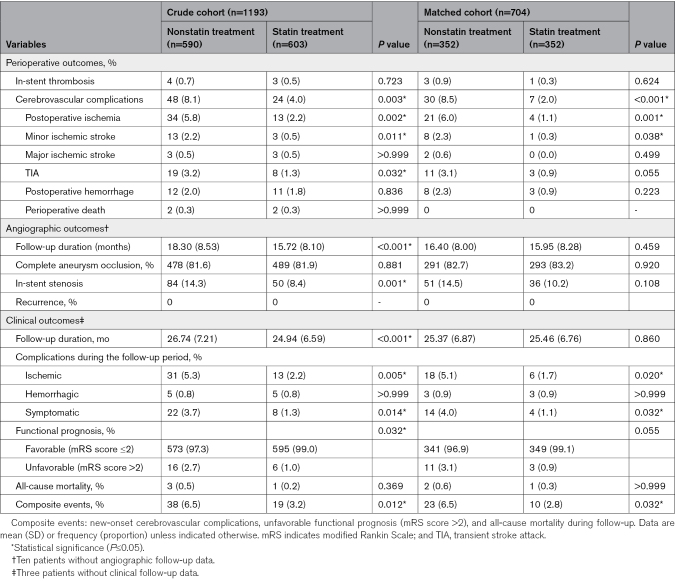
Perioperative and Follow-Up Outcomes

Angiographic follow-up data were obtained for 1183 patients (99.2%), with an average duration of 17.00±8.41 months. The complete occlusion rates were comparable between the statin and nonstatin groups (81.9% versus 81.6%, *P*=0.881). However, the statin group exhibited a significantly lower rate of ISS (8.4% versus 14.3%, *P*=0.001). In the PS-matched cohort, statin therapy showed a nonsignificant trend toward reduced ISS incidence (10.2% versus 14.5%, *P*=0.108).

A total of 1190 patients (99.7%) participated in the clinical follow-up, and the mean follow-up duration was 25.83±6.96 months. The rate of favorable functional prognosis was higher in the statin treatment group than in the nonstatin treatment group (99.0% versus 97.3%), although the difference was not significant (*P*=0.203). Interestingly, we found lower rates of ischemic complications (2.2% versus 5.3%, *P*=0.005) and composite events (3.2% versus 6.5%, *P*=0.012) in the statin treatment group, compared with the nonstatin group. Moreover, after PS-matched analysis, a significant between-group difference in ischemic complications was found.

Furthermore, we conducted a subgroup analysis of outcomes based on different durations of atorvastatin use (3–30 days versus 30–90 days versus ≥90 days). Despite the various preoperative durations of atorvastatin use, the results showed no significant statin-use-duration–based difference in treatment outcomes (Table S1).

### Analysis Stratified by Baseline LDL-Cholesterol Levels

The analysis of the relationship between statin therapy and clinical outcomes was stratified based on baseline LDL-cholesterol levels, and we specifically compared patients with levels <2.59 mmol/L to those with levels at or above this threshold (Table 3). The incidence of each type of perioperative complication was lower in the patients with lower LDL levels. Multivariable adjustment for key covariables revealed that statin treatment may provide a protective effect for patients with higher baseline LDL-cholesterol levels against the development of cerebrovascular complications (odds ratio, 0.371 [95% CI, 0.195–0.705]; *P*=0.002) and postoperative ischemia (odds ratio, 0.239 [95% CI, 0.100–0.570]; *P*=0.001). Although a similar protective trend was noted among patients with lower baseline LDL-cholesterol levels, it lacked statistical significance (*P* values for interaction were 0.671 and 0.226, respectively).

**Table 3.**
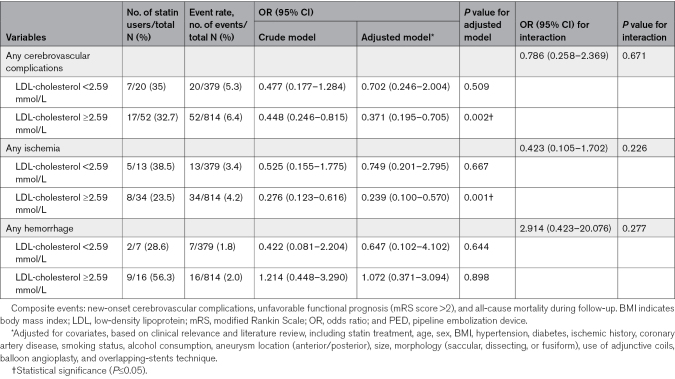
Stratification of the Risk of Post-PED Implantation Perioperative Complications According to the LDL-Cholesterol Level

**Figure 2.**
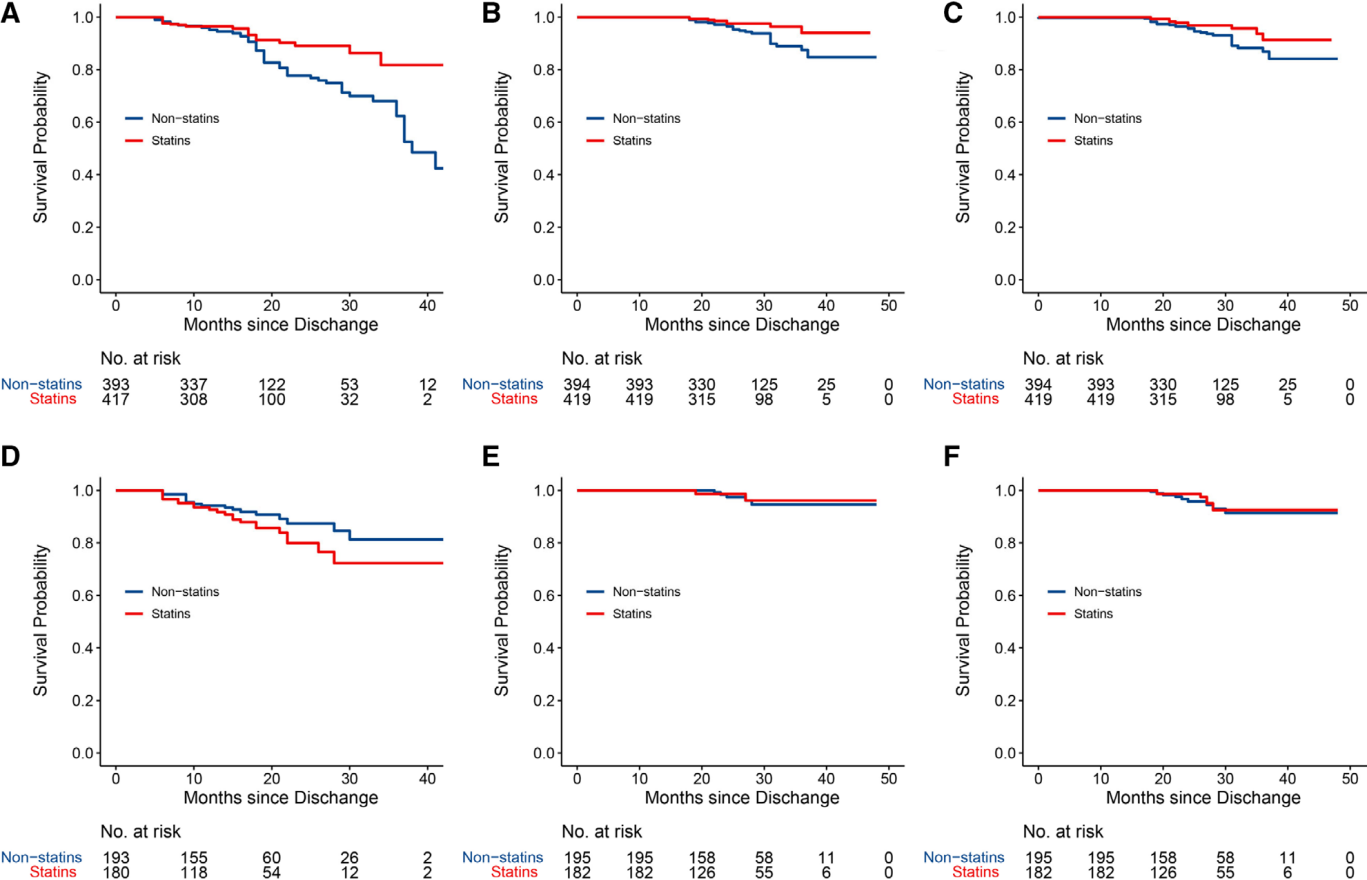
**Primary outcomes after pipeline embolization device (PED) implantation with statin treatment. A**, The incidence of in-stent stenosis in participants with baseline LDL (low-density lipoprotein)-cholesterol ≥2.59 mmol/L after PED implantation, compared with those who did and did not receive statin treatment during follow-up (*P*=0.002, log-rank test). **B**, Survival without ischemic complications after PED implantation in participants with baseline LDL-cholesterol ≥2.59 mmol/L (*P*=0.023, log-rank test). **C**, Survival without composite cerebrovascular events after PED implantation in participants with baseline LDL-cholesterol ≥2.59 mmol/L (*P*=0.038, log-rank test). **D**, Incidence of in-stent stenosis in participants with baseline LDL-cholesterol <2.59 mmol/L after PED implantation, compared with those who did and did not receive statin treatment during follow-up (*P*=0.130, log-rank test). **E**, Survival without ischemic complications after PED implantation in participants with baseline LDL-cholesterol <2.59 mmol/L (*P*=0.770, log-rank test). **F**, Survival without composite cerebrovascular events after PED implantation in participants with baseline LDL-cholesterol <2.59 mmol/L (*P*=0.650, log-rank test).

In the higher baseline LDL-cholesterol levels group, the incidence of ISS was lower for patients on statin treatment versus patients who were not on statin treatment during follow-up (hazard ratio, 0.433 [95% CI, 0.269–0.698]; *P*=0.001), as was the incidence of ischemic complications (hazard ratio, 0.315 [95% CI, 0.135–0.733]; *P*=0.007) and composite events (hazard ratio, 0.402 [95% CI, 0.193–0.838]; *P*=0.015; Table 4; Figure 2A through 2C). However, statin treatment showed no benefit in terms of angiographic outcomes in participants with baseline LDL-cholesterol <2.59 mmol/L (Figure 2D). Although the protective effect of statin treatment showed trends in clinical outcomes in patients with lower baseline LDL-cholesterol levels, the difference was not statistically significant (Figure 2E and 2F; *P* values for interaction were 0.009, 0.381, and 0.415). Furthermore, no significant benefit of statin treatment was observed for aneurysmal occlusion in either higher or lower baseline LDL-cholesterol groups (Figure S2).

**Table 4.**
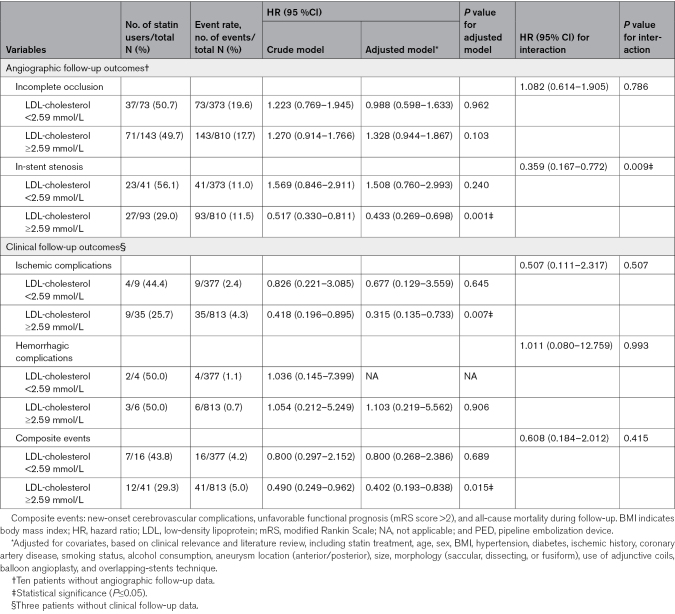
Stratification of the Risk of Post-PED Implantation Unfavorable Angiographic Follow-Up Outcomes According to the LDL-Cholesterol Levels

### Sensitivity Analysis

To validate the robustness of our model, we conducted matching analyses using inverse probability of treatment weighting. In the inverse probability of treatment weighting group, statin therapy was effective in reducing the occurrence of perioperative ischemic complications, ISS, follow-up ischemic events, and improving functional outcomes (Table S2). Furthermore, we applied the same methods for multivariable analyses to the entire cohort and similar result is confirmed by a subgroup-based PS-matched cohort (Table S3 and S4). The results revealed that statin treatment significantly reduced (all *P* value for interaction >0.05) the incidence of perioperative ischemia (odds ratio, 0.416 [95% CI, 0.211–0.821]; *P*=0.011), ISS (odds ratio, 0.397 [95% CI, 0.232–0.667]; *P*=0.001), and follow-up ischemic complications (hazard ratio, 0.364 [95% CI, 0.148–0.895]; *P*=0.028).

### LDL-Lowering Effect or Pleiotropic Effects of Statin

A total of 506 participants (42.5%) had data available regarding LDL-cholesterol levels during the follow-up period (16.78±9.47 months). To investigate whether the improved outcomes were mediated by the lipid-lowering effects of statins or their pleiotropic effects, we performed stratified analyses of LDL-cholesterol levels during the follow-up period. As shown in Table S5, regardless of statin treatment, no statistical differences were observed in outcomes based on lipid control status, except for a significantly lower composite event rate in participants with LDL-cholesterol controlled below 1.8 mmol/L (1.2% versus 6.6%, *P*=0.032). Furthermore, Tables S6 and S7 show that statin treatment improved patient outcomes, irrespective of LDL-cholesterol control status (both stratified by thresholds of 1.8 mmol/L and 2.59 mmol/L), particularly in preventing the incidence of ISS and ischemic complications.

## Discussion

In our comprehensive analysis of 1193 patients with IAs treated with PED, we conducted a meticulous evaluation of specific statin medication classes, dosages, and individual LDL-cholesterol levels. To ensure comparability, we used PS matching to equilibrate baseline characteristics. Our study revealed that statin therapy correlated with a significant decrease in perioperative complications, a pronounced effect on ischemic incidents, and superior angiographic and clinical outcomes. Although the interaction analysis was not statistically significant, subgroup analyses showed a clinically meaningful effect. The benefits of statins were more evident in patients with higher baseline LDL-cholesterol levels (≥2.59 mmol/L). Although the results of subgroup analysis should be interpreted with caution, this finding potentially has significant clinical relevance. This suggests that statin therapy is associated with better clinical and angiographic outcomes in patients who receive flow-diverter stent treatment, especially those with elevated LDL-cholesterol. This observation parallels the established benefits of statins in-stent procedures for lesions across various vascular territories, including the coronary, peripheral, and cerebrovascular circulations.

The clinical benefits of statin treatment are well established in carotid artery stenting, percutaneous coronary intervention, and abdominal aortic aneurysm repair.^9,10,24^ Previous investigations, which suggested that statin therapy exerts no significant influence on outcomes post-PED implantation, were limited by the omission of detailed statin classifications or dosing regimens, and they encompassed a modest cohort of statin-treated patients.^13,22^ Crucially, the absence of serum cholesterol data obscured the correlation between serum lipid profiles and clinical outcomes. Although the pathological characteristics of ischemic complications and ISS after flow diversion may differ fundamentally from the pathophysiology of atherosclerosis, our primary goal was not only to explore the lipid-lowering effects of statins but also to investigate their pleiotropic effects (ie, vasodilatation, platelet inhibition, anti-inflammatory function, reduced rates of neointimal hyperplasia). In the subgroup analyses stratified by lipid levels, the predominantly negative *P* values for interactions suggest that the treatment effects of statins may be consistent regardless of baseline LDL levels. This implies that the effects observed in different lipid strata are likely similar to those in the overall population, which indicates that statin therapy can generally improve outcomes after PED treatment. Thus, it is imperative to conduct further studies to determine whether the beneficial effects of statin therapy are exclusive to patients presenting with higher baseline LDL-cholesterol levels or whether these benefits are universal among all individuals who have undergone PED procedures.

A major concern with PED treatment is safety, with a non-negligible risk of postoperative ischemia.^3,25^ Considering that a higher mesh density in the PED and stent implantation may induce platelet activation, thrombosis, and inflammation within the vessel wall,^26^ ischemic events are more important peri-procedural complications during PED treatment than during traditional embolization. Statins may improve perioperative complications due to their pleiotropic effects, even before a significant reduction in serum cholesterol levels, with benefits occurring within 24 hours of initiation.^6^ Owing to their traditional lipid-lowering effects as well as pleiotropic anti-inflammatory, anticoagulation, and platelet inhibition properties, statins may serve as a reliable secondary prevention measure after PED treatment in patients with higher LDL-cholesterol levels. Although the low-cholesterol group did not experience a statistically significant benefit from statin treatment regarding perioperative complications, statin users exhibited a tendency towards reduced perioperative complications (3.3% versus 6.7%). Thus, further randomized controlled trials are ongoing to clarify the role of statins in patients with IAs who have undergone FD treatment and to identify specific subgroups that may benefit most from statin therapy, which may provide additional valuable insights in the future (URL: https://www.clinicaltrials.gov; Unique identifier: NCT06308952).

The improved long-term clinical prognosis of statin users could potentially be explained to some extent by the pleiotropic effects on the vessel walls, such as neuroprotection, improved collateral flow, and anti-inflammatory effects.^6,27^ Furthermore, statins alter cardiovascular risk by lowering LDL-cholesterol and modestly increasing high-density lipoprotein cholesterol levels.^28^ In pathological research, the ISS commonly coexists with intimal thickening, smooth muscle cell proliferation, and inflammatory cells.^29–32^ After the initial statin intake, a full therapeutic effect was achieved in 4 to 6 weeks. Therefore, the pleiotropic effects of the statin treatment regimen in our cohort (for at least 6 months) may have a protective effect against these lesions. Some studies have suggested that statin treatment could reduce the risk of vascular events in ischemic diseases, even in patients with lower LDL-cholesterol levels.^33,34^ The predominantly negative *P* values for interactions in our subgroup analyses suggest that the treatment effects of statins may be consistent regardless of baseline LDL levels. In addition, a recent prospective study found evidence of a slightly increased risk of hemorrhagic stroke events with LDL-cholesterol-lowering therapies when baseline LDL-cholesterol levels were already low.^35^ In the present study, however, we did not observe a statistical difference in new-onset hemorrhage between statin users and nonstatin users during the long-term clinical follow-up. Moreover, our stratified analysis showed that statin treatment improved outcomes regardless of LDL-C control status, with no significant differences in outcomes between different lipid control thresholds. Different from the emphasis on using high-intensity lipid-lowering in the Western population, the guideline for lipid management in our country recommended the use of low–moderate-intensity therapy.^5,23,36^ Therefore, randomized controlled trials are necessary to further investigate the effects of lipid management and dose-dependent statin therapy on outcomes after PED treatment in participants with different LDL-cholesterol levels.

In patients with cardiovascular disease, statin treatment has improved neointimal coverage of drug-eluting stents and reduced rates of neointimal hyperplasia.^37^ In another retrospective analysis of 94 patients, Flores-Milan et al^14^ found that statin treatment was the only protective factor against ISS after PED implantation. However, the generalizability of these results may be limited due to the small sample size. Thus, to date, high-quality evidence regarding whether statin therapy can improve the incidence of ISS is lacking. Our results showed that the incidence of ISS was 11.3% in the present study, and statin treatment was predictive and protective of ISS in patients with higher LDL-cholesterol levels at admission. One of the underlying mechanisms could be the crucial role of vascular wall inflammation and endothelial dysfunction in the pathogenesis of ISS.^29–32^ Thus, the anti-inflammatory and improvement in endothelial function associated with statin use may improve outcomes after PED implantation.^36,38^ However, the pleiotropic effect of statins may be masked if the target arteries do not exhibit an inflammatory response or significant endothelial dysfunction in patients with low baseline LDL-cholesterol levels. Therefore, longer follow-up or larger sample sizes may be needed to validate the generalizability and stability of this finding. Stenosis within the PED is a relatively rare but complex progression that is poorly understood and has been rarely described in the literature.^4,13^ Therefore, the association between existing dyslipidemia and the subsequent development of ISS is worth exploring in the future using a randomized controlled design.

Despite the widespread use of PED in the endovascular treatment of IAs, the key mechanism for aneurysm occlusion remains unclear. It is possible that flow-diverting effects of PED lead to thrombus formation and eventual aneurysm sac occlusion.^39^ However, statins’ pleiotropic effects, including anticoagulant and antiplatelet actions, may impede clot formation. Conversely, studies indicate that PED acts as a scaffold for intimal growth, promoting aneurysm occlusion.^40^ Furthermore, statins ameliorate endothelial dysfunction and improve vascular smooth muscle cell proliferation by upregulating the expression and activity of endothelial NO synthase.^41^ These conflicting perspectives underscore the multifactorial nature of aneurysm occlusion. Notably, the effects of statins observed in IAs from experimental models may not directly translate to clinical settings. Given the emphasis on flow diversion and lipid management, further research on statin therapy is warranted.

Dual antiplatelet therapy is a cornerstone in managing patients undergoing PED implantation. Although some studies suggest that statins may reduce the efficacy of antiplatelet agents such as clopidogrel or ticagrelor, there is no robust clinical evidence or established guidelines explicitly recommending against their combined use in vascular disease.^42–46^ In our study, we conducted platelet function testing (using thromboelastography or light transmission aggregometry) for all patients before FD implantation to assess the effectiveness of antiplatelet agents. Patients exhibiting clopidogrel resistance were switched to ticagrelor and retested for antiplatelet aggregation function, ensuring an optimized antiplatelet regimen. Therefore, none of the patients in our cohort experienced inadequate platelet inhibition due to potential interactions between statins and antiplatelet drugs, thus ensuring the efficacy and safety of PED implantation. However, as antiplatelet regimens remain a critical focus in FD treatment, these pharmacological interactions warrant further exploration in the field of IAs.

Although being the first study to investigate the association between LDL-cholesterol levels and statin treatment on the outcomes of patients who underwent PED implantation in a propensity-matched cohort, our study has some limitations. First, because of the lack of randomization, there is a potential for bias, in that patients treated versus those not treated with statins after hospitalization may be systematically different. Second, we did not assess whether the participants were responsive to statin therapy and the pharmacological testing of interactions between statins and antiplatelet agents, causing them to be unequally exposed to the effects of statins on the outcomes of PED. Third, the proportion of follow-up information on LDL-cholesterol levels is relatively low, despite lipid profile is not a routine laboratory examination conducted during follow-up after flow-diversion treatment. Fourth, we did not analyze the impact of statin type and intensity on outcomes, which warrants further investigation in future studies. Furthermore, although this was a prospective multicenter study, the patient cohort was restricted to the Chinese population; studies involving other racial and ethnic groups are needed to confirm the generalizability of our results. Finally, registry-based retrospective studies have some inherent limitations. Unmeasured confounding factors could not be excluded despite rigorous propensity adjustments to mitigate baseline imbalances. To substantiate these findings and inform clinical practice, additional targeted interventional trials and large-scale prospective studies are needed and will be instrumental for refining the therapeutic management strategies in patients undergoing FD stent therapy.

In conclusion, our study demonstrated that atorvastatin therapy is significantly associated with reduced perioperative complications, particularly in mitigating ischemic events, and confers superior angiographic and clinical outcomes. The interaction analyses revealed that the therapeutic efficacy of statins may be consistent regardless of baseline LDL levels, despite the statistically significant effect in patients with higher baseline LDL-cholesterol levels (≥2.59 mmol/L) as compared with those with lower levels (<2.59 mmol/L). These findings underscore the clinical significance of statin therapy in the context of flow-diverter stent treatment and highlight its potential to improve outcomes, which provides insights for clinical decision-making. The implications of our study warrant further exploration through targeted clinical trials to confirm the role of statins in vascular interventional procedures.

## Article Information

### Acknowledgments

The authors thank Professor Peihua Cao for the guidance with the statistical analysis and Professor Daniel Raper for the guidance with the statistical analysis and revision of the manuscript.

### Author Contributions

All authors contributed to the manuscript, satisfying the International Committee of Medical Journal Editors guidelines for authorship. Drs Liu and Duan are guarantors of integrity of entire study. Dr Feng contributed to funding, study supervision, and critical revision of the manuscript. Dr Huang contributed to study design and drafted the manuscript. Drs Tong and Wen analyzed and interpreted the data. Drs Zhu, Huang, and Lin contributed to methodology validation. Dr Huang, Dr Yuan, Dr Xu, Dr Ma, R. Ge, C. Li, and Dr Peng contributed to literature research and data acquisition. Drs Su, Zhang, Li, and Guo contributed to management and critically revised the study outcomes. Drs Guo, Liu, and Duan conceptualized the study, funding, study supervision, and critical revision of the manuscript.

### Sources of Funding

This research project was supported by the Natural Science Foundation of China (grant nos. 82201427, 82071332, and 82171290) awarded to Drs Feng, Guo, and Liu, and the Foundation of National Health Commission Capacity Building and Continuing Education Center (grant number GWJJ2022100102) awarded to Dr Duan.

### Disclosures

None.

### Supplemental Material

Supplemental Methods

Figure S1–S2

Table S1–S7

STROBE Checklist

## Supplementary Material


